# On the Treatment of Cystic Goitre

**Published:** 1892-07-09

**Authors:** 


### SURGERY.

### ON THE TREATMENT OF CYSTIC GOITRE.

In the treatment of cystic goitres we have a choice between treatment by tapping with or without injections, and by extirpation.

As regards tapping there are many modifications. The injections of iodine and of iron solutions have found many advocates. Some practitioners simply tap, but if nothing more be done the cyst very frequently refills. The cyst may be tapped and a drainage tube inserted, with somewhat better results. Dr. Woakes* has described a method of employing chromic acid, which has given very happy results. It was suggested to him by his experience about six years ago, when he had under treatment a woman, aged fifty, for fibro-cystic goitre, involving the right lobe of the thyroid body. The cyst, which extended to within an inch of the angle of the jaw, had been tapped and injected with a concentrated solution of tannin, with the result of securing apparent obliteration of the cavity. She continued under observation, but after an interval of six months it became evident that the cyst was refilling. In the course of a few weeks the contents, consisting of a thin purulent fluid, found an exit through the cicatrix left after the original tapping, with the consequent formation of a sinus, through which the offensive fluid continually issued. Thus was established the exact nuisance which the method originally adopted was designed the obviate. The difficulty of treating the cyst though the sinus was increased by the fact that during the lengthened absences of the patient, a portion of its now tortuous course passed through recently-formed and still growing hypertrophic gland tissue. Under these circumstances a probe, previously dipped for three inches of its length into a saturated solution of chromic acid, was passed through the sinus into the cyst cavity. This was done several times in three successive weeks, then, after an interval of non-attendance of some five weeks' duration, a brown, tough material was found protruding from the wound. Traction in this drew out a rolled-up mass of the same substance, which, when spread out, had the appearance of leather, and was about the size of the palm of one's hand. It was undoubtedly the wall of the cyst, altered and probably destroyed by contact with the chromic acid, and thereafter got rid of as described, and it is needless to state that after this deliverance the cystic part of the trouble was ended. The author relates several other cases in which the use of chromic acid was successful in obtaining a cure, but further confirmation of the utility of such a procedure is afforded by Mr. W. R. H. Stewart†, who has treated seven cases of cystic goitre with eminently satisfactory results. The tumours were tapped in the usual manner, and the contents washed out. After all hæmorrhage had ceased, the saturated chromic acid solution was applied with a carrier through the cannula to the walls of the cyst. Six of the seven cases healed rapidly after from two to three applications, but the seventh and second of the series resisted for a long time all attempts, and it was not until three months had passed and some half-a-dozen applications had been made that the tumour disappeared. But neither in this nor in any of the other cases was there a bad symptom. The length of time taken by the last case to heal is attributed by the author to the fact that there was a considerable amount of hæmorrhagic oozing, which, to a certain extent, neutralised the action of the acid. It is therefore advisable to see that hæmorrhage is, as much as possible, arrested before applying the acid.

Iodoform injections have been employed by Kapper‡ in the treatment of cystic goitre. The solution used contained one part of iodoform to seven of ether and seven of olive oil, this liquid being injected with an ordinary hypodermic syringe. The latter is introduced about an inch into the tumour (at several places where the growth is large), fifteen to twenty minims being injected at a time. As a rule, the injection is repeated every four to six days, but on several occasions daily treatment was resorted to without any unpleasant sequelæ. Kapper employed these injections in fifteen cases, and in every one of them a reduction of about three to four inches in the circumference of the neck took place. The patients throughout were able to continue at their usual avocations.

* *Lancet*, June 21st, 1890. †*Lancet*, Dec. 19th, 1891.

‡ *British Medical Journal*, Aug. 29th, 1891.

The question of enucleation and extirpation of cystic goitres may have to be considered, for some cystic goitres are altogether too large to admit of any satisfactory result from injections. The advantages of enucleation are the less danger of cachexia and less risk of wounding the nerves or great vessels, but on the other hand, as Dr. Berry remarked in his lectures at the Royal College of Surgeons, there is greater likelihood of recurrence than when the goitre is partly excised.

A very large cystic goitre under Bornis, of Tubingen* is the largest struma ever operated upon. The tumour was double as thick as the diameter of the trunk, and reached down to the umbilicus. It was removed by extra capsular extirpation. The patient was cured in a short time. The tumour was a cyst weighing ten pounds. Charters Symonds† has had very favourable results from extirpation of cystic and solid goitres. His experience in an earlier case led him to see the advantages of first searching for the capsule and then enucleating. In several cases this was done and the growths were removed without loss of any blood and with great facility. As a rule, at most these small vessels required ligature. All the patients recovered with primary union, and most required but one dressing subsequently to that made at the operation. Briefly his method may be described as follows: Wherever the tumour may be situated, in every case make a median incision, for it gives more room and leaves the smallest cicatrix, and when the deep layer of fascia is divided the largest growths can be brought to the median line. Then lay bare the thin cyst wall and dissect it off the gland; if the fibrous wall be followed closely no bleeding or further trouble need be feared.

If the white glistening wall of a solid tumour, or the bluish wall of a cystic one is not seen at once, then the edge of the gland must be sought and raised up till the capsule is seen. If a dissection is commenced outside this, severe bleeding may be encountered. In the case of a cyst, Mr. Symonds advocates opening it early, and after sufficient of the wall has been exposed, to secure with forceps, and dissecting back the thyroid, just as in ovariotomy. By this means the operation can be performed through a smaller opening, and the resulting scar is slight. In Mr. Symonds' cases all the cysts contained in the wall a variable amount of gelatinous glandular material, which showed the usual veins lined by cortical or columnar epithelium, and this structure was exactly the same as in the solid forms. The author regards as cases suitable for operation those in which the growth is localised, well defined, and limited to one side. In none of his cases was there more than the one tumour, but multiplicity is not to be considered to negative the operation. He maintains that excision gives more speedy recovery, and is freer from danger than any other method.

We cannot deny that the methods first alluded to, viz., the simpler operation of tapping and injection, is attended with special dangers as well as special advantages, and may even become a very severe procedure and attended with the formation of abscess and a resulting hectic.

* *Journ. Laryngal.* Aug., 1891. † *Lancet*, Dec. 21st, 1889.

